# Vasa Vasorum in Saphenous Vein for CABG: A Review of Morphological Characteristics

**DOI:** 10.21470/1678-9741-2023-0045

**Published:** 2023-08-07

**Authors:** Andrzej Loesch

**Affiliations:** 1 Research Department of Inflammation, Centre for Rheumatology and Connective Tissue Diseases, Division of Medicine, University College London, London, United Kingdom

**Keywords:** Vasa Vasorum, Pericytes, Basement Membrane, Saphenous Vein, CABG

## Abstract

This short article discusses selected scanning electron microscope and transmission electron microscope features of vasa vasorum including pericytes and basement membrane of the human saphenous vein (SV) harvested with either conventional (CON) or no-touch (NT) technique for coronary artery bypass grafting. Scanning electron microscope data shows the general damage to vasa vasorum of CON-SV, while the transmission electron microscope data presents ultrastructural features of the vasa in more detail. Hence there are some features suggesting pericyte involvement in the contraction of vasa blood vessels, particularly in CON-SV. Other features associated with the vasa vasorum of both CON-SV and NT-SV preparations include thickened and/or multiplied layers of the basement membrane. In some cases, multiple layers of basement membrane embrace both pericyte and vasa microvessel making an impression of a “unit” made by basement membrane-pericyte-endothelium/microvessel. It can be speculated that this structural arrangement has an effect on the contractile and/or relaxing properties of the vessels involved. Endothelial colocalization of immunoreactive inducible nitric oxide synthase and endothelin-1 can be observed (with laser confocal microscope) in some of the vasa microvessels. It can be speculated that this phenomenon, particularly of the expression of inducible nitric oxide synthase, might be related to structurally changed vasa vessels, *e.g.,* with expanded basement membrane. Fine physiological relationships between vasa vasorum endothelium, basement membrane, pericyte, and perivascular nerves have yet to be uncovered in the detail needed for better understanding of the cells’specific effects in SV preparations for coronary artery bypass grafting.

## INTRODUCTION

The superiority of saphenous vein (SV) graft harvested by no-touch (NT) technique for coronary artery bypass grafting (CABG) described by Souza^[1]^ compared to commonly applied conventional (CON) harvesting procedures was highlighted in the recent special edition of the Brazilian Journal of Cardiovascular Surgery (2022;37[Special 1] 1-78; for Editorial, see Gomes et al.^[2]^). Various anatomical and physiological factors may account for the success of NT-SV as coronary graft^[1,3]^. The preservation of the functioning vasa vasorum supplying blood to the wall of the SV as coronary graft seems particularly profound, as is observed at the time of NT-SV graft implantation during CABG^[4]^. This harvesting technique ensures that the intact wall of NT-SV graft receives circulating factors including oxygen, ensuring good physiological start for the graft. This initial anti-ischemic approach may have a great positive impact on the physiological adaptation of the graft to the new hemodynamic condition. Taking into account the importance of the blood supply to the SV as CABG, this review presents in the first instance some morphological details of the vein vasa vasorum; this is followed by the discussions of various structural aspects of the SV vasa vasorum system including its pericytes and basement membrane.

## GENERAL FACTS ABOUT VASA VASORUM IN SAPHENOUS VEIN

**Fig. 1:**
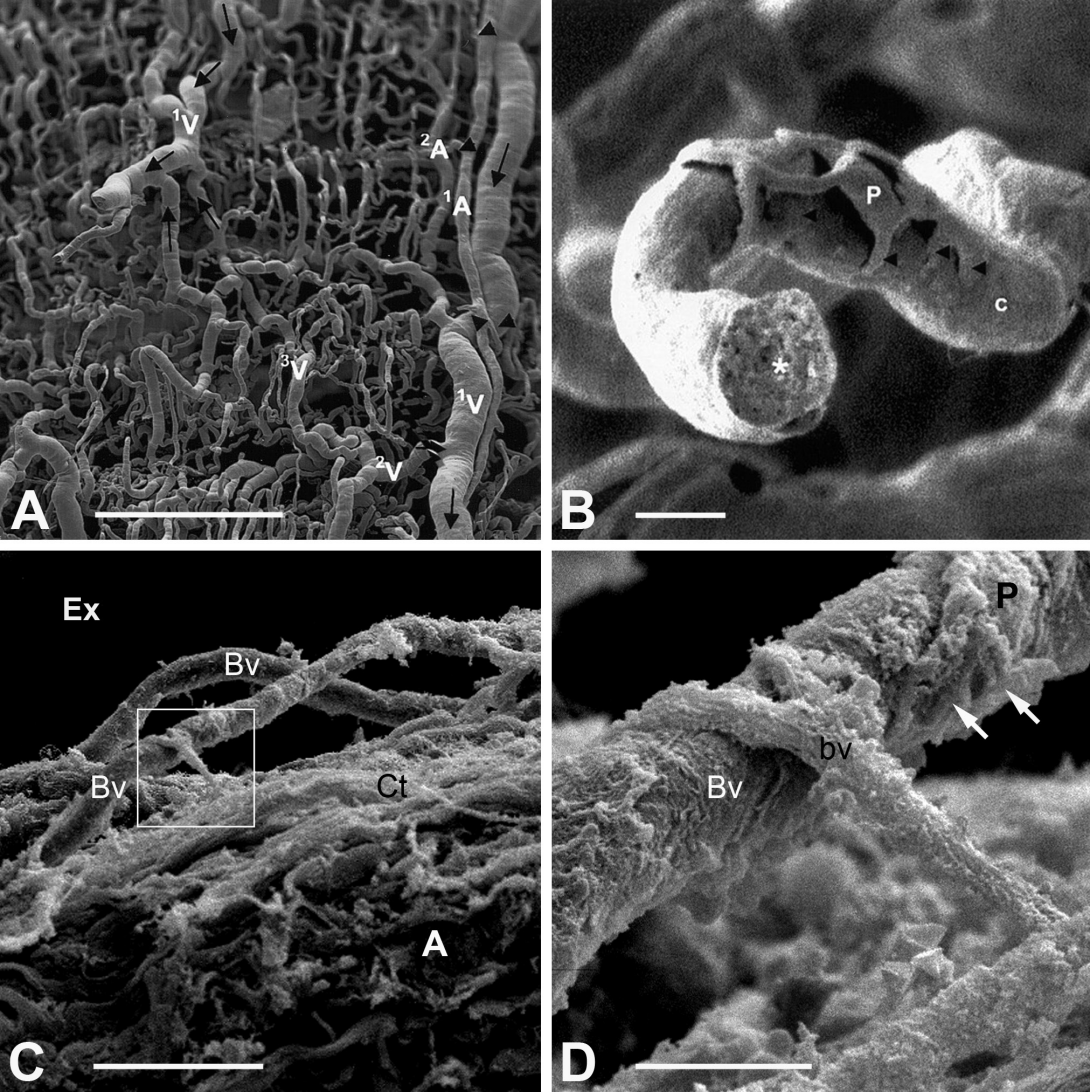
Scanning electron microscopy (SEM) of vascular corrosion casts (A and B) and standard SEM preparations (C and D) of saphenous vein (SV) harvested for coronary artery bypass grafting. A) Note a complex pattern of vasa vasorum and the presence of the first (1V)-, second (2V)-, and third (3V)-order veins, as well as the second-order arteries (arrowheads); vessels supply the first- (1A) and second (2A)-order arteries. Arrows indicate direction of blood flow. B) Note a pericyte (P) embracing a capillary (c). Arrowheads mark lateral processes of the pericyte, and the asterisk marks the internal structure of the resin cast. C) Side view of conventional SV demonstrates damaged adventitia (A) with vasa vasorum blood vessels (bv) exposed to the external environment (Ex). Framed area is magnified in D. D) Magnified fragment of microvessel from C shows P-like structure and its processes (arrows). Also note an anastomosing structure — possibly a small donating microvessel (bv). Bars: A) 0.5 mm; B and D) 10 µm; C) 50 µm. It is acknowledged that A) and B) images are from Lametschwandtner et al.^[7]^, 2004; and C) and D) are from Vasilakis et al.^[8]^, 2004. Ct=connective tissue.

The historical data and the correct usage of the Latin term “vasa vasorum”, broadly defined as blood vessels feeding the wall of larger blood vessels (*e.g.,* human SV), has recently been highlighted^[5,6]^. Since SV is commonly used for CABG, it is reasonable to assume that any new finding concerning this vessel is important and therefore should be accurately noted. This would not only improve the accuracy of vascular research, but also contribute to our better understanding of why the application of certain clinical procedures should, or perhaps should not, be applied. To add to the general discussion about vasa vasorum and SV for CABG, here are shown some data from scanning electron microscope (SEM) as well as from transmission electron microscope (TEM); a brief data from a laser confocal microscope (LCM) is included. It should be pointed out that except for two SEM images (Figures 1A and B) of vasa vasorum of SV (for details, see Lametschwandtner et al.^[7]^), the SEM and TEM images presented here are from a research (re-visited here) carried out by the author and his students (for details, see Vasilakis et al.^[8]^, Ahmed et al.^[9]^) on CON-SV and NT-SV preparations harvested from patients during CABG at Örebrö University Cardiothoracic Surgery, Sweden. Due to various health issues, patients undergoing CABG with the SV as a coronary graft may not always have an anatomically “perfect” SV, but the vein can nonetheless be acceptable as a graft. It may come as no surprise, therefore, that some fine anatomical details of SV, including the details of its vasa vasorum vessels, are unknown during CABG surgery. In this context, the sections below attempt to present some known and perhaps less known histological details relevant to SV vasa vasorum and CABG application.

### Vasa Vasorum of Saphenous Vein: Scanning Electron Microscope

Elegant studies of resin corrosion casts of human SV examined at SEM level revealed a complex spatial arrangement of vasa vasorum system in the vein wall, consisting of arterial and venous vascular network including microvessels and associated pericytes^[7,10,11]^. According to Lametschwandtner et al.^[7]^, this complex vasa vasorum in SV closely follows the longitudinally oriented connective tissue fibers in the adventitia and the circularly arranged vascular smooth muscle cell layers within the outer media. These features, therefore, reflect the importance of the vasa vasorum in supplying blood to the SV wall. As an example, Figures 1A and B demonstrate SEM images of vasa vasorum and pericytes in corrosion casts of SV (nonskeletonized) harvested for CABG, where complexity and abundance of the vasa vasorum can be observed. In contrast, Figures 1C and D present SEM images of vasa vasorum in the CON-SV harvested for CABG, where pedicle removal and vein distention caused damage to the vein original architecture. These SEM images clearly show damage and even exposure of some of the vasa vessels in CON-SV preparations. For more SEM details of SV harvested for CABG, see Vasilakis et al.^[8]^.

### Vasa Vasorum of Saphenous Vein: Transmission Electron Microscope

**Fig. 2:**
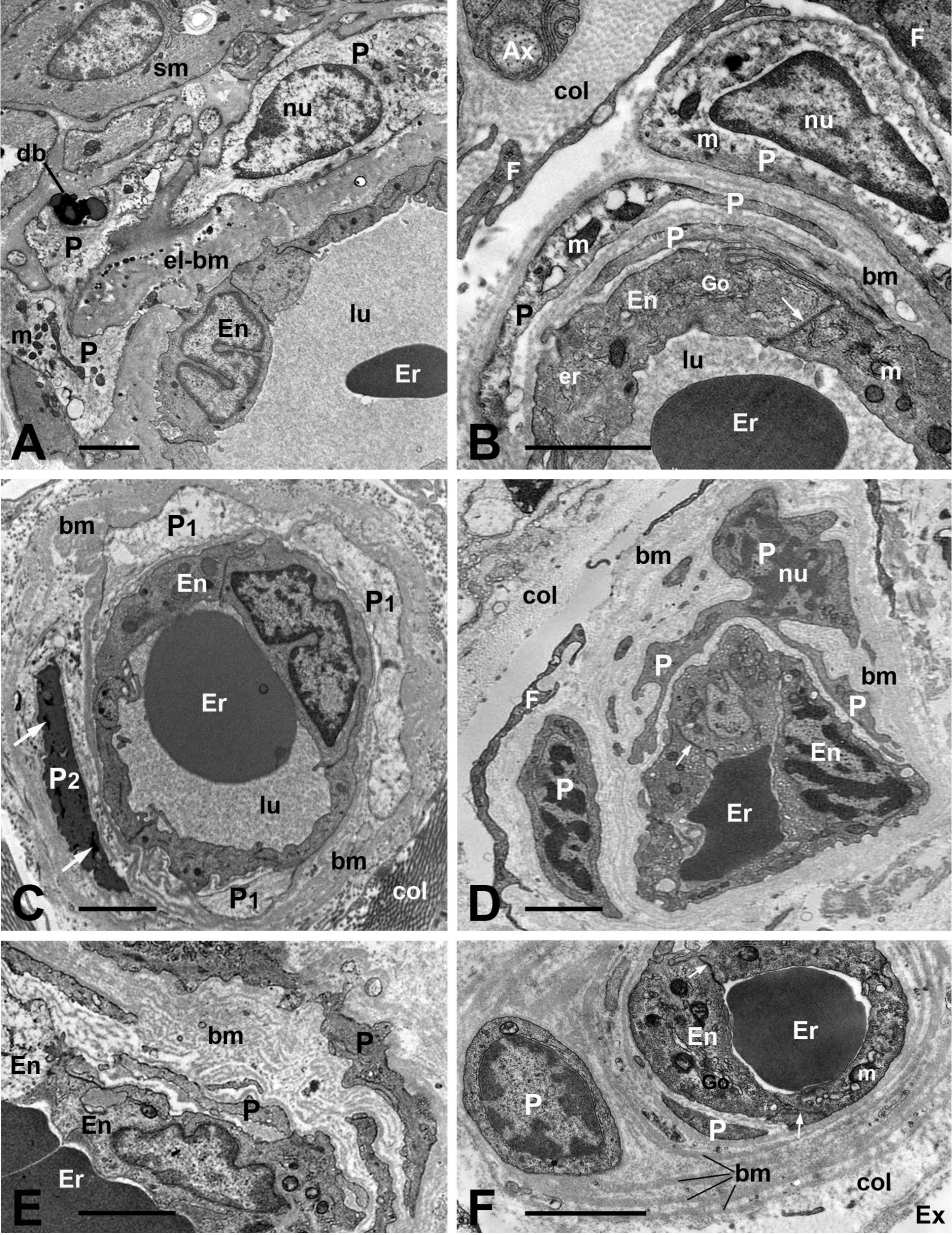
Transmission electron microscopy features of adventitial vasa vasorum in no-touch saphenous vein (SV) (A-C) and conventional SV (C-E) harvested for coronary artery bypass grafting. A) An arteriole shows open lumen (lu), erythrocyte (Er), endothelial cells (En), vascular smooth muscle (sm), and pericytes (P) with a light cytoplasm containing mitochondria (m) and dense bodies (db). B) A venule with the open lu displays Er, En, P, and their processes and basement membrane of about 30 nm – 130 nm thick. Also note inter-endothelial junction (arrow), Golgi complex (Go), endoplasmic reticulum (er), fibroblast profiles (F), collagen (col), and an axon (Ax) of perivascular nerves. C) A venule with open lu is surrounded by P processes with either light (P1) or dense (P2) cytoplasm; the later contains small aggregates of glycogen-like structure (arrows). Note varying thickness of the basement membrane. D) A remaining adventitia shows a contracted venule clumping an Er; note the P soma with prominent nucleus, and P processes embracing the vessel; arrow=inter-endothelial junction. The vessel is surrounded by multiple parallel strands/layers (each 30 nm – 70 nm thick) of the basement membrane. E) A fragment of contracted venule; note subendothelial space containing multiple undulating layers (each ~ 50 nm – 80 nm thick) of basement membrane. F) Note a structural “unit” established by multiple ring-like layers of the basement membrane encircling a capillary and P; the thickness of individual rings varies between 80 nm and 260 nm. Also note well-preserved En and interendothelial junctions (arrows); the edge of damaged adventitia and the external environment (Ex) can be seen. Bars:A-F) 2 µm. It is acknowledged that A) and F) images are from a Loesch unpublished study; B), D), and E) are from Ahmed et al.^[9]^, 2004; and C) is from Dreifaldt et al.^[4]^, 2011. nu=nucleus; el-bm=elastic lamina-basement membrane.

To view more structural characteristics of the vasa vasorum in SV harvested for CABG, TEM images are presented here (Figure 2). Figures 2A to C show TEM examples of adventitial vasa vasorum blood vessels in NT-SV preparations, where the adventitia was preserved; hence no vein stripping and distention were applied. In such NT-SV specimens, the vasa vasorum vessels seem relaxed — having lumen open (Figures 2A to C). In contrast, in CON-SV preparations the lumen in many vasa microvessels becomes contracted (Figure 2D). More TEM details concerning structural features of SV harvested for CABG can be found in earlier publications describing vasa vasorum endothelium, vascular smooth muscle, and perivascular autonomic nerves^[9]^. Most recently, TEM features of endothelial cells of NT-SV have been highlighted in the context of cell preservation in relation to the graft patency^[12]^. Some TEM data on pericytes in vasa vasorum of SV harvested for CABG are presented below.

### Vasa Vasorum Pericytes: Transmission Electron Microscope

Here pericytes are clearly seen as cellular components of the SV vasa vasorum blood vessels including arterioles, venules, and capillaries (Figures 2A to D). Not only morphological characteristics of pericytes can be seen, but also the relationship of the cells with other vascular components of the vasa vessels. In general, variations of the pericyte shape can be noted, from elongated- to spindle-shaped (Figure 2C), or the cell perinuclear region appears bulging towards abluminal site and in fact the cell is contracted (Figure 2D). Pericytes also express variations in the electron-density of the cytoplasm and the quantity of cytoplasmic organelles and structures. For example, pericytes and/or their processes in Figures 2A and B display rather light cytoplasm, Figure 2C shows both light and dense appearance of pericyte processes, while in Figure 2D the pericyte cytoplasm is of a moderate density.

### Relation: Vasa Vasorum, Pericytes, and Basement Membrane: Transmission Electron Microscope

A common feature of blood vessels including vasa vasorum of SV is the presence of the basement membrane, which is well-known to be a part of the extracellular matrix^[13]^. The electron-dense layer of the basement membrane, known as the lamina densa, is usually 30 nm - 100 nm thick (or thicker depending on the tissue) and clearly visible at TEM level. In this review, the term basement membrane is used to indicate mostly electron-dense lamina densa. In human SV vasa vasorum, lamina densa is usually about 50 nm – 80 nm thick (own unpublished observations); it is present at abluminal site of the endothelium as well as around pericytes and the vascular smooth muscle cells. A demarcation between the basement membrane and elastic lamina is not always clear, for instance, in the vasa vasorum arterioles (Figure 2A). In Figure 2B, more or less as a typical appearance of the basement membrane supporting endothelium and pericyte can be observed. However, some variations and/or abnormalities as to the shape, thickness, and multiplication of the basement membrane of vasa vasorum can be seen (Figures 2C to F). The most interesting feature is the appearance of multiple rings of the basement membrane encompassing a microvessel together with its pericyte (Figure 2F); thickness of the rings may vary, *e.g.,* from 80 nm to 260 nm.This whole structure can be seen as a “unit”, comprising: a vessel, endothelium, pericyte, and layers/rings of the basement membrane (Figure 2F). It can be speculated that in such cases this complex ring structure of the basement membrane is less elastic, hence “resistant” to vessel contraction and/or relaxation. On the other hand, thickened or multilayered basement membrane can be seen in contracted vessels of CON-SV (Figure 2D).

### Vasa Vasorum and Basement Membrane: Knowns and Speculations

Observations of enlarged basement membrane in human SV are not new. For example, enlarged basement membrane appearing either as a thick lamina densa or a multi-layered structure has previously been reported at SV luminal endothelium of smokers. This enlargement has been attributed to fibronectin accumulation^[14]^. Whether the enlarged basement membrane in SV vasa vasorum presented in the current review is related to a fibronectin accumulation or other components of extracellular matrix remains to be explored. The subject is interesting as basement membrane has complex roles. Apart from its well-known adhesion/supporting function and its role in storage of growth factors and cytokines, basement membrane constitutes diffusion barrier, hence its implication in permeability and flow of nutrients, metabolites, and signaling molecules^[15]^. As there are natural structural variations of SV in patients undergoing CABG^[16]^, some variations in the properties of the basement membrane cannot be ruled out. In various genetic diseases there are mutations in basement membrane constituents and subsequent abnormal function of the basement membrane^[15]^. Anyhow, the phenomenon of the enlargement — thickening of the basement membrane — has been a subject of debate in relation to pathology of various vascular beds, *e.g.,* in renal glomerulus in diabetic neuropathy^[17]^. In psoriatic patients with kidney glomerulonephritis, a multiplication of the basement membrane in peritubular capillaries coincides with renal allograft rejection^[18]^. Abnormal basement membrane displaying loose association with endothelial cells and pericytes has been revealed in tumor blood vessels, where these changes seem to be related to the dynamic nature of the cells^[19]^. In the retinal microcirculation of diabetic dogs, for instance, the thickness of the basement membrane is remarkable, compared to normal, and appears as an amorphous or fine fibrillary layer. This coincides with the loss of pericytes and vascular smooth muscle cells^[20]^.

As for the human SV, the question arises as to the means of communication between the endothelial cells and pericytes, and also perivascular autonomic nerves^[21]^ if the distances between these cells are obstructed by the progressive increase of the basement membrane. In other words, is it possible that the vasoactive agents essential for the communication between endothelium pericyte and perivascular nerves can pass through the enlarged basement membrane layers without degradation to act on specific receptors? The possibility cannot be excluded that in such conditions endothelial cells are the major players in delivering vasoactive agents required for the control of vascular tone, particularly if the effects of ischemia or shear stress are of concern^[22,23]^. It should be remembered that dissection of SV from the leg de facto denervates the vein during CABG harvesting; the state of functioning of the remaining vasa vasorum innervation is unknown^[21]^. Usually the neural-endothelial interactions are important, if not essential, in the control ofvasculartone^[23]^,butthere are some exceptions, for instance, human umbilical artery and vein. These vessels are not innervated so it is likely that the regulation of vascular tone there is largely dependent on endothelium-deriving vasoactive agents influencing feto-placental blood flow^[24]^. In fact, a number of vasoactive agents were immunocytochemically identified in umbilical endothelial cells^[25]^ including vasoconstrictor endothelin-1 (ET-1 ) and nitric oxide synthase (NOS), the enzyme involved in the synthesis of vasodilator nitric oxide (NO)^[24,26]^.

**Fig. 3:**
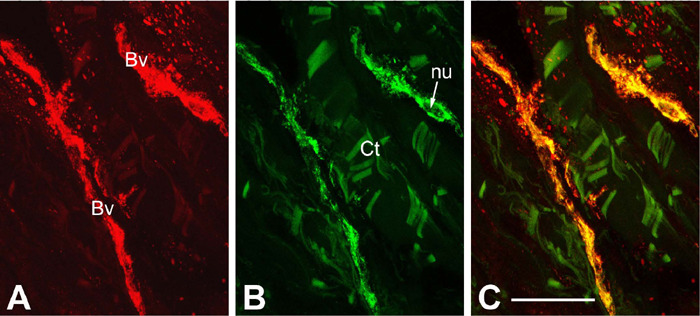
Confocal microscopy of 30 µm frozen cross-sections through the adventita of no-touch saphenous vein (~ 30 min to harvesting for coronary artery bypass grafting) co-immunolabelled for inducible nitric oxide synthase (iNOS) and endothelin-1 (ET-1). A) iNOS-immunoreactivity (red) in vasa blood vessels (Bv); B) ET-1-immunoreactivity (bright green) in the same vessels; C) the vessels show patter of endothelial co-localization of iNOS and ET-1 (yellow). Bar: 50 µm. Note the main steps of immunoprocedure involved: (1) fixation with 4% paraformaldehyde; (2) incubation with a rabbit polyclonal antibody to iNOS (Santa Cruz Biotech) and a mouse monoclonal antibody to ET-1 (Peninsula Labs); (3) incubation with a goat anti-rabbit immunoglobulin G Mexa Fluor® 568 (to detect iNOS) and a goat ant i-mouse immunoglobulin G Mexa Fluor® 488 (to detect ET-1) (both from Molecular Probes); (4) embedment in Citifluor; and (5) examination at a laser microscope: Leica DMRBE with SPZ confocal head. The images were collected at 1.5 µm intervals and then merged as maximal projection]. Images are from a Loesch unpublished study. Ct=adventitial connective tissue; nu=endothelial cell nucleus.

In human SV, a rich presence of immunoreactive endothelial NOS (eNOS) can be observed both in the vein luminal endothelium as well as in the endothelium of the vein vast vasa vasorum system, suggesting the importance of NO for vein physiology^[4,27,28]^. But due to pedicle removal and adventitial damage, there is a significant depletion of vasa vasorum and immunoreactive eNOS in CON-SV graft preparations for CABG. Clearly, this kind of damage affects the blood supply to the graft wall, which in turn may have a detrimental effect on the graft patency^[28]^. In relation to the expression of NOS by SV, here Figure 3 shows an observation of co-expression of inducible NOS (iNOS) and ET-1 in vasa vasorum of NT-SV The role of co-expression of iNOS and ET-1, which here seem to be related to the endothelium, is unknown at this stage. But it is possible that this co-expression of iNOS and ET-1 concerns the vasa vessels affected by a thick or multiplied layers of basement membrane, therefore, where the communication between endothelium, pericyte, and perivascular nerve might be obstructed. In such cases, the possibility exists that the endothelium undertakes dominant signaling and vasomotor roles. It has to be stressed that the vasa vasorum endothelium, both in CON-SV and NT-SV preparations presented here, showed normal appearance of intracellular organelles and structures including Golgi complex, endoplasmic reticulum, and mitochondria, and where endothelial intercellular junctions are in morphological order. Usually, expression (or increased expression) of iNOS and subsequently increased NO production can be linked with immunocytotoxicity and pathological conditions^[29]^. Expression of iNOS and also a co-expression of iNOS and ET-1 have previously been reported in structurally damaged vascular smooth muscle of CON-SV, while at the same time Western blot analysis of the media of the vein showed increased iNOS and ET-1 levels^[30]^. The physiological role of co-expression of iNOS and ET-1 in SV damaged vascular smooth muscle is unclear, as is this phenomenon in the vasa vasorum endothelium observed here. But, in general terms, the interrelationship between NO and ET-1 is well-recognized, where ET-1 is a component of NO signaling, and where NO can tonically inhibit ET-1 vasoconstrictor action, in particular in pathophysiological circumstances^[31]^. No images of the expression of vasoactive agents by pericytes of SV vasa vasorum are demonstrated here. Nonetheless, to better understand morphological details of electron microscope findings including pericytes, and the possible impact these details may have on the SV harvested as CABG, an inclusion of general facts about pericytes seems justified.

### GENERAL FACTS ABOUT PERICYTES

There is a mutual association between vasa vasorum and pericytes. It is well-known that pericytes are mural cells in the vascular system. However, identification of the cells is not always easy as there is no one specific marker of the cells. Usually, identification of pericytes is based on positive staining for the neural/glial antigen 2 and platelet-derived growth factor receptor-β, but depending on the study, staining for some other factors as well might be necessary^[32,33]^. Nonetheless, as part of the vascular system, including the vasa vasorum, these cells may influence a variety of tissues and organs^[33]^. Vascular pericytes are morphologically heterogeneous, with varying biochemical, immunocytochemical, and molecular constitutions; these features allow the cells to be involved in a variety of physiological and pathophysiological events^[34,38]^. These include participation of pericytes in vessel formation, which in pathology contributes to the development of metastasis and tumour vascularization^[39]^. Pericytes are implicated in diabetic microangiopathy, hypertension, and multiple sclerosis^[40]^. Importantly, pericytes also regulate the blood-brain barrier^[41]^ and contribute to pathogenesis of vascular cognitive impairment and dementia^[42]^.

One of the most striking characteristics of vascular pericytes is their ability to contract and relax, thus contributing to the mechanisms of local control of microvascular blood flow, as it has been shown in the rat retina and cerebellum^[43]^; for more details on pericytes and their role in cerebral microvessels, see indicated publications^[36,41,42,44,45,46]^. This contractile property of pericytes depends on the presence of specific contractile proteins and receptors in the cells, and on how these react to various endogenous and exogenous agents^[43,47]^. Among contractile proteins expressed by pericytes are the alpha-smooth muscle actin (a-SMA) (this is mostly detected in pericytes of pre-capillary arterioles and post-capillary venules), myosin, tropomyosin, and desmin^[48,50,51]^. The expression of these proteins may vary between the microvascular beds or even within the segments of the same bed^[34,48,49]^. In general, the smooth muscle-related pericytes (transitional) that express α-SMA are known to be contractile in nature and hence engaged in the regulation of the capillaries blood flow, whereas the pericytes that lack α-SMA may have a different functional role^[34]^. Examples of vasoactive agents that might cause pericyte to contract, hence a microvessel, include uridine-5’-triphosphate, adenosine -5’-triphosphate, and noradrenaline^[43]^. In reality, a variety of vasoactive substances, including acetylcholine, histamine, serotonin, angiotensin-ll and ET-1, may stimulate (through respective receptors) the pericytes to contract^[35,46,52,53,54,55,56]^.

The contractile role of pericytes is especially significant in the cerebral microvessels, where they take part in local mechanisms regulating the blood supply to the brain tissue^[43]^. Myocardial microvessel pericytes may play a similar important role^[57]^. However, in some tissues, like the human SV, the role of pericytes and vasa vasorum might seem “less important” than in other blood vessels, for instance, those in the brain or the heart. Consequently, during CON harvesting of SV for CABG, little attention is usually given to the preservation of the vein vasa vasorum; the adventitia is damaged due to vein stripping and distention resulting in removal and/or a severe injury of the vasa vasorum blood vessels (Figures 1C and D).This is in contrast to NT harvesting procedures, where all elements of the wall of SV are preserved, including the vein vasa vasorum^[1,4]^.

### Clinical Potential for Vasa Vasorum Pericytes

One of the features of pericytes of the vasa vasorum of human SV is the expression of a transmembrane phosphoglycoprotein — clusterofdifferentiation 34(transmembrane phosphoglycoprotein) (CD34) —, which is a common progenitor cell marker; importantly, these CD34-positive pericytes are negative for the endothelial marker — cluster of differentiation 31 (transmembrane highly glycosylated protein) (CD31)^[32]^. Here, it has been shown that CD34-positive pericytes are able to give rise to highly proliferative cells expressing pericyte/mesenchymal antigens and stem cell marker Sox2; the cells also possess clonogenic and multi-lineage differentiation capacities. Therefore, such pericytes can be used in regenerative processes, *e.g.,* for the treatment of damaged heart following an acute ischemic event due to coronary artery disease^[58,59,60]^. In fact, the CD34-positive but CD31-negative pericytes isolated from human SV vasa vessels can be selectively cultured in order to obtain “saphenous vein-derived progenitor cells” (SVPs)^[32]^. When such SVPs are placed in the vascular pool of the cardiac peri-infarct zone of allogenic recipient, they integrate with endothelial cells, and through paracrine mechanisms release/secrete a number of factors, including angiopoietin-1 and vascular endothelial growth factor-A, hence promoting reparative angiogenesis, cardiomyocyte survival, and inhibition of interstitial fibrosis^[59]^. Clearly, there is a potential for SVPs to be used for the treatment of ischemic cardiovascular diseases. In this context, the role of pericytes in SV is important as these cells might affect the course of physiological and pathophysiological events, participate in the reparative mechanisms, and potentially contribute to the graft patency.

More recently, partially due to the Coronavirus disease 2019 (COVID-19) pandemic, increased attention has also been given to the association of angiotensin-converting enzyme 2 (ACE2) with the vasculature, including microvessels. This has also raised the possibility of the severe acute respiratory syndrome Coronavirus 2 entering pericytes via ACE2, acting as a virus receptor, altering pericyte contractile properties, and subsequently affecting the vasa vasorum system^[61,62]^.The vasa vasorum in human SV is rich in immunoreactive ACE2^[63]^. Immunohistochemical examinations of the internal mammary and radial arteries from patients undergoing CABG also revealed a rich presence of ACE2 in the neointima and media of healthy and diseased arteries^[64]^. Strikingly, however, ACE2 has not been detected in the endothelium of the lumen of the arteries, but only in the vasa vasorum and newly formed angiogenic vessels. The possibility of expression of ACE2 in human endothelial cells is disputable. Recent analysis of the ACE2 ribonucleic acid sequencing of human vascular cells suggests an abundant presence of ACE2 in pericytes while ACE2 is scarce in endothelial cells^[65]^. Thus, the possibility of pericytes being implicated in the viral infections and alteration of human microvessels, including that of the vasa vasorum of SV, is highly likely. This possible scenario of altered vasa in SV in post-COVID-19 patients might be significant, though difficult to judge at this stage.

## CONCLUSION

Data available so far suggest that SV pericytes influence the physiology of the vasa vasorum, hence impacting on the condition of the vein wall. Based on ultrastructural characteristics, there are images of pericytes suggesting their ability to adjust the lumenal diameter of vasa microvessels, *e.g.,* constricting the vessels during CABG CON harvesting. It is not clear at this stage, however, if the enlargement of the basement membrane has an impact on communication between the endothelium, pericytes, and perivascular nerves, and whether this affects contractile properties of vasa microvessels. Nonetheless, the importance of the preservation of vasa vasorum in SV used as a coronary graft seems important as has previously been discussed in detail^[66]^. There are still plenty of unknowns, *e.g.,* the distribution of subclasses of pericytes and how these functionally relate to the complexity and variety of the vasa vasorum microvessels. The task is difficult partially because no universal marker for pericytes is available. In their elegant review article on pericytes, Dessalles et al.^[67]^ state “*The field of pericyte mechonisms and mechonobiology remains in its infancy”.* This statement seems very true in relation to the role of pericytes in human SV, in particular, in the context the cells’ physiological responses when the vein is harvested to be used as CABG.

## Abbreviations, Acronyms & Symbols

α-SMA: Alpha-smooth muscle actin
ACE2: Angiotensin-Converting enzyme 2
CABG: Coronary artery bypass grafting
CD31: Cluster of differentiation 31 (transmembrane highly glycosylated protein)
CD34: Cluster of differentiation 34 (transmembrane phosphoglycoprotein)
CON: Conventional
COVID-19: Coronavirus disease 2019
eNOS: Endothelial nitric oxide synthase
ET-1: Endothelin-1
iNOS: Inducible nitric oxide synthase
LCM: Laser confocal microscope
NO: Nitric oxide
NOS: Nitric oxide synthase
NT: No-touch
SEM: Scanning electron microscope
SV: Saphenous vein
SVPs: Saphenous vein-derived progenitor cells
TEM: Transmission electron microscope
